# Transgenic increases in seed oil content are associated with the differential expression of novel *Brassica*-specific transcripts

**DOI:** 10.1186/1471-2164-9-619

**Published:** 2008-12-19

**Authors:** Nirmala Sharma, Maureen Anderson, Arvind Kumar, Yan Zhang, E Michael Giblin, Suzanne R Abrams, L Irina Zaharia, David C Taylor, Pierre R Fobert

**Affiliations:** 1 National Research Council Canada, Plant Biotechnology Institute, NRC, 101 Gymnasium Place, Saskatoon, SK S7N 0W9, Canada

## Abstract

**Background:**

Seed oil accumulates primarily as triacylglycerol (TAG). While the biochemical pathway for TAG biosynthesis is known, its regulation remains unclear. Previous research identified microsomal diacylglycerol acyltransferase 1 (DGAT1, EC 2.3.1.20) as controlling a rate-limiting step in the TAG biosynthesis pathway. Of note, overexpression of *DGAT1 *results in substantial increases in oil content and seed size. To further analyze the global consequences of manipulating *DGAT1 *levels during seed development, a concerted transcriptome and metabolome analysis of transgenic *B. napus *prototypes was performed.

**Results:**

Using a targeted *Brassica *cDNA microarray, about 200 genes were differentially expressed in two independent transgenic lines analyzed. Interestingly, 24–33% of the targets showing significant changes have no matching gene in *Arabidopsis *although these represent only 5% of the targets on the microarray. Further analysis of some of these novel transcripts indicated that several are inducible by ABA in microspore-derived embryos. Of the 200 *Arabidopsis *genes implicated in lipid biology present on the microarray, 36 were found to be differentially regulated in DGAT transgenic lines. Furthermore, kinetic reverse transcriptase Polymerase Chain Reaction (k-PCR) analysis revealed up-regulation of genes encoding enzymes of the Kennedy pathway involved in assembly of TAGs. Hormone profiling indicated that levels of auxins and cytokinins varied between transgenic lines and untransformed controls, while differences in the pool sizes of ABA and catabolites were only observed at later stages of development.

**Conclusion:**

Our results indicate that the increased TAG accumulation observed in transgenic *DGAT1 *plants is associated with modest transcriptional and hormonal changes during seed development that are not limited to the TAG biosynthesis pathway. These might be associated with feedback or feed-forward effects due to altered levels of DGAT1 activity. The fact that a large fraction of significant amplicons have no matching genes in *Arabidopsis *compromised our ability to draw concrete inferences from the data at this stage, but has led to the identification of novel genes of potential interest.

## Background

Genomics has emerged as a powerful tool for crop improvement [reviewed in [1-3]]. In particular, the development of high-throughput methods for genome analysis, such as DNA microarrays, serial analysis of gene expression, and massively parallel signature sequencing, have enabled the scientific community to unravel molecular mechanisms underlying plant phenotypic traits of economic importance. Functional genomics studies have identified potential candidate genes and regulatory factors for adaptation, yield and quality traits, which could be introgressed at an accelerated rate into elite germplasm by marker-assisted breeding or directly engineered into economically important crop plants. In case of major crops such as rice, where whole genome sequence is available for analysis, breeding programs have already benefited from advances made in functional genomics [1]. For minor crop species with limited available sequence information, comparative genomics has been employed to facilitate functional genomics studies to improve crop performance [2].

In combination with molecular genetics, genomics research have given us unprecedented knowledge of the biochemical pathways for seed oil biosynthesis and metabolism, and of the genes encoding the enzymes that mediate the reactions [reviewed in [4-6]]. In most plant species, seed oil accumulates primarily in the form of triacylglycerol (TAG) which serve as an energy reserve for the germinating seed. Fatty acids synthesized in plastids are sequentially incorporated onto a glycerol backbone in the endoplasmic reticulum through a series of acyl-CoA-dependent acylations commonly known as the Kennedy pathway. First, *sn*-glycerol-3-phosphate is acylated by the action of glycerol-3-phosphate acyltransferase (GPAT; EC 2.3.1.15) and subsequently lyso-phosphatidic acid acyltransferase (LPAAT; EC 2.3.1.51) to produce lysophosphatidic acid (PA). The PA is then dephosphorylated by phosphatidate phosphatase (PAP; EC3.1.3.4) to form *sn*-1,2- diacylglycerol (DAG) which is finally acylated by diacylglycerol acyltransferase (DGAT; EC 2.3.1.20) to give TAG.

In the traditional Kennedy pathway, DGAT is the only enzyme that is exclusively committed to TAG biosynthesis using acyl-CoAs as substrate. DGAT activity is relatively low compared to the activities of other enzymes in pathway [7,8] and the DGAT substrate (DAG) accumulates in developing seeds [9]. These findings suggest that DGAT may represent a restriction point in seed oil formation. This hypothesis is substantiated by genetic and transgenic analyses showing that seeds from *Arabidopsis *plants with mutant alleles of *AtDGAT1 *accumulate substantially less TAG than the wild-type [10,11] while over expression of DGAT1 increases oil content and seed size in transgenic *Arabidopsis *and *Brassica napus *[12-15]. Biochemical analysis of the developing transgenic seeds has confirmed an increase in microsomal DGAT-specific activity and a decreased ratio in DAG:TAG compared to wild-type, consistent with a greater proportion of DAG being converted to TAG.

Although many of the biochemical steps in TAG biosynthesis are known, its regulation remains unclear. In addition to DGAT, the mitochondrial pyruvate dehydrogenase complex (mtPDC) has been identified as a key regulator of seed oil accumulation. Silencing of mtPDC kinase (PDCK), a negative regulator of mtPDC, results in substantial increases in oil content and seed size in *Arabidopsis *and *Brassica *[16,17]. Mitochondrial PDC acts to regulate the turnover of pyruvate into the fatty acid precursor acetyl-CoA. Recent studies have revealed the importance of transcriptional control for seed oil bioassembly. For example, altering levels of key transcription factors, including the *Arabidopsis *WRINKLED1 (WRI1), GLABRA2, LEAFY COTYLEDON1 (LEC1), LEC2, ABSCISIC ACID INSENSITIVE3 (ABI3), FUSCA3 (reviewed in [18]) or soybean DNA BINDING WITH ONE FINGER4 (DOF4) and DOF11 [19] has significant effects on seed oil levels. Although the specific contribution of most of these transcription factors to seed oil biosynthesis remains to be determined, WRI1 has been implicated in the control of genes encoding enzymes of late glycolysis and *de novo *fatty acid biosynthesis, including mtPDC and pyruvate kinase [20]. Furthermore, LEC2 is a positive regulator of WRI1 [20]. Comparison of gene expression in seeds of two near-isogenic lines (NILs) of *B. napus *displaying a 10% difference in seed oil content has confirmed that differences in TAG levels is associated with the differential accumulation of transcripts for numerous genes [21]. Using subtractive suppression hybridization, these authors identified 36 differentially regulated genes, including those encoding enzymes involved in lipid-related processes and transcription factors.

Numerous genetic and physiological studies have demonstrated that the plant hormone abscisic acid (ABA) plays a critical role in the regulation of seed maturation, including seed oil accumulation [reviewed in [22]]. ABA induces the accumulation of certain genes encoding enzymes of lipid bioassembly pathways, for example, those involved in erucic acid synthesis [23]. Recently, ABA and hydroxylated ABA metabolites were shown to increase oil content of *B. napus *microspore-derived embryos (MDEs) and to induce the expression of the lipid marker genes *FATTY ACID ELONGASE1 *(*FAE1*) and *OLEOSIN2*, suggesting a role for these compounds in regulating TAG biosynthesis [24]. Combined with the finding that some key transcription factors involved in lipid metabolism are under the influence of ABA signaling [reviewed in [18,25]], the data suggest a link between ABA and transcriptional regulation of seed oil biosynthesis. However, the correlation, if any, between profiles of major plant hormones including ABA, auxins and gibberellic acid (GA) during seed maturation and oil levels has not been examined previously.

We are interested in exploiting transgenic plants displaying elevated levels of seed oil to further our understanding of TAG biosynthesis and its regulation. Although it is possible that the phenotype observed in transgenic plants overexpressing DGAT1 is due to solely to increased activity of this one enzymatic step, it is conceivable also that altering DGAT1 activity results in secondary metabolic or regulatory effects that contribute to the phenotype. For example, Jako et al. [12] speculated that increases in DGAT1 activity may lower the size of the acyl-CoA pools, thereby signaling a need for enhanced fatty acid synthesis. As a first step in ascertaining possible secondary effects associated with transgenic DGAT1 overexpression, we performed comprehensive transcript and hormone profiling of *B. napus *prototypes transformed with the *Arabidopsis DGAT1 *gene under the control of the strong seed-specific promoter napin. Our analysis identified modest transcript and hormonal effects in the transgenic plants, including some general as well as lipid metabolism-specific transcriptional changes. Of note, many of the transcripts displaying large differences in expression were novel and appear to be specific to *Brassica*.

## Results

### *DGAT1 *overexpression increases seed oil content

This study analyzed transgenic *B. napus *(*cv*. Quantum) prototypes expressing the *Arabidopsis DGAT1 *coding region under the control of the strong seed-specific napin promoter. The structure of *Arabidopsis *DGAT1 is highly homologous to the subsequently reported putative *B. napus *DGAT1 at both the nucleotide (92%) and the deduced amino acid (90%) levels [26]. In our experience, the activities of the *B. napus *and *Arabidopsis *enzymes in microsomal fractions assayed *in vitro *are of the same order of magnitude. Transgenic *B. napus *plants overexpressing either *DGAT1 *genes also display very similar phenotypes with respect to seed oil content and seed size [14]. Thus, either gene is suitable for the purposes of this study and the *Arabidopsis *gene was selected primarily out of convenience, as it was the first to be isolated and suitable transgenic prototypes were in a more advanced stage of development at the onset of this project.

**Figure 1:**
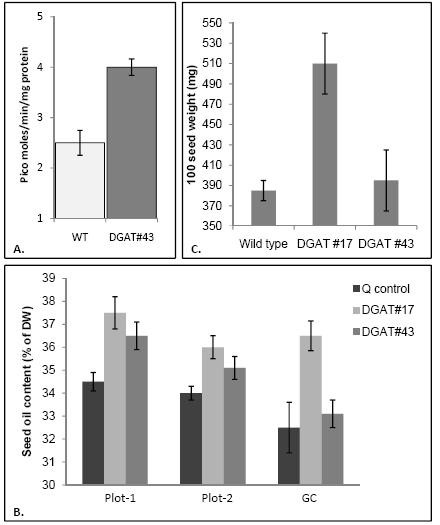
**Biochemical analysis of transgenic seeds**. **(A) **DGAT activity in mid-maturation seeds of non-transformed control (Q-CON) and DGAT#43 (napin:*AtDGAT1 *T_4 _transfomants *B. napus cv*. Quantum) grown in growth cabinets. Values are the average of three determinations ± SE. **(B) **Oil content of non-transformed control and two napin:DGAT1 transgenic lines of *B. napus cv*. Quantum. Values represent averages ± SE. Plot-1 and Plot-2, data from a 2006 confined field trial; GC, data from seeds amplified in a controlled growth chamber. **(C) **Average 100-seed weight of untransformed *cv*. Quantum and transgenic lines amplified in a growth chamber. Values represent averages ± SE.

Two independent transgenic lines were analyzed. One line, DGAT#17, was chosen because it accumulated substantially more seed oil and produced larger seeds than untransformed controls, while the second, DGAT#43, was chosen because it displays more modest seed-related phenotypes. Detailed molecular, biochemical and physiological characterization of DGAT#17 will be published elsewhere [14]. It contains 4–5 copies of the T-DNA, accumulates >100-fold more *AtDGAT1 *transcript, and possesses 10 times more DGAT activity than untransformed controls. Line DGAT#43 contains two T-DNA insertions, expresses lower levels of the transgene than DGAT#17 (data not shown) and possesses about 1.6 times more DGAT activity than untransformed *cv*. Quantum controls (Figure 1A).

Oil content was determined in the two independent transgenic lines in growth chambers and in confined field trials and seed weight was measured in growth chambers only. When amplified in growth chambers, mature seeds (T_4 _generation) of DGAT#17 showed oil content increases of about 3–4% dry weight (Figure 1C) over untransformed *cv*. Quantum, and seed weight increases of 20–29% (Figure 1B). In confined field trials (2006), DGAT#17 exhibited 3–6% increments in seed oil content (Figure 1C); this trend was consistent with a previous drought year (2003) where a 3% increment in oil content was observed compared to the untransformed control (data not shown). DGAT#43 displayed more modest increases in seed oil content over untransformed seeds, ranging from an 0.6% increase in growth cabinets to 1–3% increases in the field (Figure 1C), but was no different from the untransformed control with respect to seed weight (Figure 1B). There was no change in the acyl composition of the TAGs in the transgenic lines (data not shown). Thus, both transgenic lines analyzed in this study have elevated seed oil content, consistent with previous reports of DGAT1 overexpression [12-15].

### Overall changes in seed transcriptome associated with transgenic expression of *DGAT*

DNA microarrays have been successfully used to examine the changes in gene expression in *Arabidopsis *mutants that are altered in lipid metabolism and seed maturation processes (e.g. [27-29]). However, the lack of available microarray platforms has limited the potential of global gene expression approaches in *B. napus*. Several studies have exploited available genomic resources based on *Arabidopsis*, a close relative of *B. napus*, but such cross-species microarray analyses are not without problem [30]. Accordingly, we recently helped validate a *Brassica *amplicon microarray representing about 10,000 unigenes which was designed using EST sequences biased for seed-derived cDNAs [31]. We exploited this microarray platform to analyze gene expression in transgenic seeds at different times during early to mid-maturation stages. The first time point analyzed, 18 days after anthesis (daa), was chosen to coincide with the onset of oil deposition, while the remaining times (22 and 26 daa) span the period of active seed oil biosynthesis [21]. At each selected stage of seed development, probes derived from mRNA extracted from transgenic seeds were co-hybridized with those derived from untransformed *cv*. Quantum, allowing accurate and reliable comparison of gene expression between the materials analyzed.

**Figure 2:**
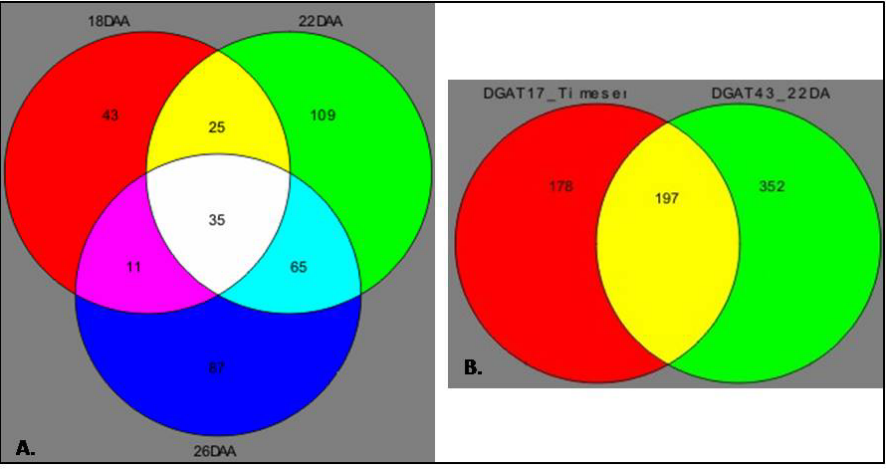
**Venn diagrams summarizing differential gene expression between untransformed *cv*. Quantum and DGAT transgenic seeds**. **(A) **Comparison of differential gene expression in seeds of line DGAT#17 at three time points during the period of TAG deposition. Genes were considered differentially expressed from *cv*. Quantum if found to be statistically significant by SAM at a false discovery rate of 5% and showing a minimum 1.5-fold change in expression from the untransformed cultivar. 18AA, 22DAA and 26DAA, seeds analyzed for gene expression 18, 22 and 26 days after anthesis, respectively. **(B) **Overlap of differentially expressed genes between the two independent transgenic lines analyzed.

Significance analysis of microarrays (SAM) with a false discovery rate of ≤ 5% and a 1.5-fold change-in-expression cut-off identified 114, 234 and 198 genes differentially expressed in the DGAT transgenic line #17 at 18 daa, 22 daa and 26 daa, respectively (Figure 2A). A substantial number of genes (212 genes) were differentially regulated at two time points and a small number (35) were detected as being differentially regulated at all three developmental time points. Overall, a total of 375 differentially regulated genes were identified. More genes were identified as being differentially expressed at 22 daa and 26 daa compared to the earlier time point (18 daa). A list of the most highly differentially regulated transcripts between untransformed *cv*. Quantum and DGAT#17 is presented in Table 1. A notable observation from this list of genes is that none are regulated in opposite directions (up- versus down-regulated) over the time course analyzed.

**Table 1:** Genes showing ≥ 2-fold change in expression between DGAT1 transgenics and untransformed control (*cv*. Quantum).

**GenBank accession #**	**Top hit in *Arabidopsis***	**Description**	**Fold change**			**Regulation**
				
			**Day18**	**Day22**	**Day26**	
CX278332	No hit	Novel	11.7	14.7	25.8	Down
CN726366	No hit	Novel	14.1	21.5	16.0	Down
EE462460	At3g09950	Similar to hypothetical protein	20.6	18.6	15.0	Down
CX279214	No hit	Novel	ND	7.1	5.5	Down
EE549605	No hit	Novel	6.0	4.9	3.2	Down
EE436000	At1g70770	Similar to unknown protein	5.2	3.21	4.2	Down
DY004194	At3g22600	Protease inhibitor/seed storage/lipid transfer protein (LTP) family protein	ND	ND	3.75	Down
EE550782	At1g73600	Phosphoethanolamine N-methyltransferase	ND	1.8	4.3	Down
EE569901	At5g56530	Similar to unknown protein	2.8	1.6	2.3	Down
EE544201	No hit	Novel	2.9	2.5	3.3	Down
EE404484	No hit	Novel	2.0	2.51	2.2	Down
CN726984	At2g43050	Pectinesterase-like protein ATPMEPCRD;pectinesterase	1.7	2.8	ND	Down
EE541726	At1g74370	Zinc finger (C3HC4-type RING finger) family protein	2.5	2.4	1.7	Down
CN726378	At5g45690	Similar to unknown protein	ND	2.3	1.7	Down
EE548935	Atmg00910	Hypothetical mitochondrial protein	ND	2.0	ND	Down
EE549536	At5g26260	Meprin and TRAF homology domain-containing protein	2.9	4.8	ND	Up
DY009332	At5g45890	SAG12 (Senescence-associated gene 12)	ND	4.5	1.7	Up
CN731950	At1g10190	Similar to unknown protein	ND	4.4	ND	Up
EE462239	At1g52400	BGL1 (Beta-glycosidase homolog 1)	4.4	2.6	ND	Up
CN729787	At3g20210	DELTA-VPE (delta vacuolar processing enzyme)	ND	4.9	ND	Up
EE569354	At3g20210	DELTA-VPE (delta vacuolar processing enzyme)	ND	4.0	1.4	Up
EE462019	At5g26280	Meprin and TRAF homology domain-containing protein	3.8	3.7	1.7	Up
CN729334	At5g59310	LTP4 (Lipid transfer protein 4)	2.5	3.8	ND	Up
CX272737	At1g04645	Self-incompatibility protein-related	ND	3.6	ND	Up
CN729331	At5g45890	SAG12 (Senescence-associated gene 12)	1.6	3.5	ND	Up
DY009689	At3g24120	Myb family transcription factor	ND	3.2	1.4	Up
CN731251	No hit	Novel	ND	3.1	1.9	Up
CN737110	At5g57920	Plastocyanin-like domain-containing protein	ND	3.0	ND	Up
DY013300	At4g37900	Glycine-rich protein	ND	2.9	ND	Up
EE541123	At4g30880	Protease inhibitor/seed storage/lipid transfer protein (LTP) family protein	ND	2.7	ND	Up
EE543120	At5g46930	Invertase/pectin methylesterase inhibitor family protein	ND	2.6	ND	Up
CX273000	At4g37900	Glycine-rich protein	ND	2.6	ND	Up
CN726334	No hit	Novel	2.5	1.9	ND	Up
CN730052	At1g71250	GDSL-motif lipase/hydrolase family protein	ND	2.4	1.7	Up
DY009919	At5g33340	CDR1(Constitute disease resistance1)	ND	2.4	2.1	Up
DY007598	At1g45130	BGAL5 (beta-galactosidase 5)	ND	2.3	1.7	Up

Fold-change values obtained using ≤ 5% False Discovery Rate (FDR) and a q-value = 0. ND, not detected by microarrays; No hit, probe sequence did not hit any gene in *Arabidopsis*.

Since a large number of transcriptional changes were observed at 22 daa in DGAT#17, this time point was selected for profiling of transgenic line DGAT#43. Comparison of DGAT#43 and untransformed *cv*. Quantum identified 549 differentially regulated genes, 197 of which were also identified as being differentially regulated at one or more points in the DGAT#17 time series analysis (Figure 2B; a full list of these genes is provided in Additional File 1). In general, genes that were detected as being differentially regulated in both transgenic lines were the ones found to be the most statistically significant by SAM and/or possessed the largest fold-change differences. There was also good agreement on the magnitude and direction of change in both transgenic lines. Of note, 31 of the 35 genes detected at all time points in DGAT#17 are among the genes differentially regulated in DGAT#43.

### Annotation and functional classification of differentially expressed genes

Since the *B. napus *genome is not fully sequenced, we relied on sequence information and the annotation available from its close relative *Arabidopsis thaliana*. Gene Ontology (GO) annotation for probes showing significant expression changes in DGAT lines were derived based on the closest hit on *Arabidopsis *chromosomes [31]. Functional classification was therefore limited to those probes for which a matching AGI was available at the time of writing this manuscript.

About 24% of the amplicons showing significant expression changes in DGAT#17 have no matching counterpart in *Arabidopsis*, other plant species, or any organism. This conclusion is based on similarity searches at both the nucleotide and protein level of the TAIR and UniProt databases, and suggests that these transcripts might be specific to *Brassica*, and hence, novel. The proportion of novel transcripts increased substantially, to 35%, when we considered only those genes found to be differentially expressed in both DGAT lines and to about 45% if we considered a 2-fold-change cut-off of the probes identified by SAM. In contrast, this classification represents only 5% of the targets on the microarray [31]. Probes for some of the novel transcripts were among the most highly differentially expressed at all the time points (Table 1). Additionally, most of the novel probes showing remarkably high level of expression changes are down-regulated (Table 1).

Besides the largest groups of genes that have unknown cellular associations and predicted to encode unknown proteins, many of the identified genes with matching relatives in *Arabidopsis *appeared to encode membrane bound products and involved in membrane-related or lipid-related processes including oil body assembly proteins (detailed below). Based on functional characterization for GO biological processes, genes in the top three represented categories were annotated as being involved in unknown functions, other metabolic processes, and other cellular processes. GO-Slim classification also identified groups of genes based on predicted molecular functions; again the largest group of genes (37.5%) belonged to the 'unknown molecular function' category followed by 'other enzyme activity' (16.5%), hydrolase activity and other binding activity. A smaller fraction of genes belonged to transferase activity and transporter activity groups. Differentially regulated genes encoding hydrolase activity listed in Table 1 include SAG12 (putative esterase), BGL1 (glycosidase), BGAL5 (galactosidase), CDR1 (aspartic proteinase), a putative pectinesterase (CN726984), and DELTA-VPE (caspase). Although, to our knowledge, none of these genes have been implicated in seed oil deposition, the DELTA-VPE is specifically expressed in seed coats where it is required for mediating programmed cell death [32]. Differentially regulated genes encoding membrane bound functions include a self-incompatibility related protein (CX272737; Table 1), plastocyanin-like domain protein (CN737110; Table 1), several tonoplast intrinsic proteins (Additional File 1), and several enzymes with lipid-related functions, such as oleosins, glucose-6-phosphatase, and a putative ketoacyl-CoA synthase.

Genes encoding lipid-related functions were present across the above classifications, prompting us to undertake a more targeted analysis of lipid-related genes. Of a total of 270 probes on the cDNA microarray corresponding to 194 *Arabidopsis *genes implicated in lipid biology [33], thirty six genes were found to be differentially regulated in DGAT transgenic lines (Table 2). Twelve of these were detected in both independent transgenic lines. Raw datasets confirmed that all probes corresponding to the 194 lipid genes produced detectable signals but the intensity was similar in the transgenic and untransformed samples rendering them non-significant, and implying that only 36 of those present on the arrays were affected by DGAT over expression. Twenty out of these 36 probes were found to correspond to *Arabidopsis *genes involved in biosynthesis, transport and/or storage of lipid suggesting that DGAT over expression has a major effect on these processes (Table 2). Included were probes encoding Oleosins – proteins that coat the surface of oil bodies [34]. *Oleosin2*, *Oleosin3 *and *Oleosin4 *were identified as being down-regulated at 22 daa but were not differentially expressed at 18 daa or 26 daa. Multiple lipid-transfer proteins (LTP) were also identified as being differentially regulated in DGAT transgenics (Table 1, Table 2). While some LTP isoforms were up-regulated, others were down-regulated. LTPs can enhance the *in vitro *transfer of phospholipids between membranes and participate in the regulation of intracellular fatty acid pools [35]. Other differentially-regulated lipid genes of note include those encoding enzymes involved in the production of acetyl-CoA; two of the three core subunits of mtPDC (DY009539 and EE543730, Table 2) responsible for the production of acetyl-CoA from pyruvate [17]; and the B subunit of ATP citrate lyase (DY010115, Table 2) which catalyzes the production of acetyl-CoA from citrate in the cytosol [36]. As mentioned in the background section, increasing mtPDC activity by antisense suppression of PDCK results in increased seed oil accumulation [16,17], while antisense suppression of ATP citrate lyase in *Arabidopsis *leads to a complex phenotype that includes reduction of seed oil levels [37].

**Table 2:** Summary of lipid-related probes differently expressed in seeds of DGAT transgenic plants.

**GO cellular process**	**GenBank accession #**	**Top hit in *Arabidopsis***	**Description**
Lipid accumulation/Storage (6)	CN733866	At3g27660	Oleosin4
	EE404224	At3g27660	Oleosin4
	CN734396	At3g27660	Oleosin4
	CN733358	At5g40420	Oleosin2
	CN725729	At5g51210	Oleosin3
	EE404495	At5g40420	Oleosin2

Lipid transport/lipid binding (7)	DY013108	At2g38540	LTP1 similar to LP1 (nonspecific lipid transfer protein 1)
	EE548798	At2g38540	LTP1 similar to LP1 (nonspecific lipid transfer protein 1)
	DY009497	At5g55410	Protease inhibitor/seed storage/lipid transfer protein family protein
	DY004194	At3g22600	Protease inhibitor/seed storage/lipid transfer protein family protein
	CN727063	At5g55450	Protease inhibitor/seed storage/lipid transfer protein family protein
	EE541123	At4g30880	Protease inhibitor/seed storage/lipid transfer protein family protein
	CN729334	At5g59310	Encodes a member of the lipid transfer protein family

Fatty acid biosynthesis (7)	EE439753	At2g47240	Long-chain acyl-CoA synthetase
	DY003951	At5g47635	Unknown protein
	CX269616	At5g35360	Biotin carboxylase subunit (CAC2)
	CX270177	At5g35360	Biotin carboxylase subunit (CAC2)
	CX268549	At3g05420	Acyl-CoA binding protein
	CX266957	At2g38040	Acetyl-CoA carboxylase
	DY010115	At5g49460	B subunit of ATP Citrate lyase

Lipid Metabolism (5)	DY009539	At1g34430	E2 subunit of Pyruvate dehydrogenase complex (PDC)
	DY004112	At2g43710	Stearoyl-ACP desaturase
	CN736133	At1g59900	E1 subunit of PDC
	EE543730	At1g06080	delta 9 acyl-lipid desaturases
	CX278600	At4g29720	Polyamine oxidase

Cuticlebiosynt hesis (2)	DY009608	At1g67730	Ketoreductase/oxidoreductase
	DY013393	At2g26250	3-ketoacyl-CoA synthase

Miscellaneous (8)	ES264646	At3g18000	N-methyltransferase-like protein
	CN728393	At3g18000	N-methyltransferase-like protein
	CN734808	At4g16155	Lipoamide dehydrogenase 2
	EE435778	At1g54100	Aldehyde dehydrogenase
	EE462569	At4g17480	Palmitoyl thioesterase
	EE550782	At1g73600	Phosphoethanolamine N-methyltransferase
	CN733236	At4g26740	Arabidopsis Seed Gene1 (ATS1)
	CN726535	At4g26740	Arabidopsis Seed Gene1 (ATS1)

Glycolipid biosynthesis (1)	EE550786	At4g00550	UDP-galactose-dependent digalactosyldiacylglycerol synthase

Functional categorization based on closest hit on *Arabidopsis *chromosome, and the annotation of corresponding genes.

Of the transcription factors implicated in regulating seed oil accumulation, only *LEC1*, which encodes a transcriptional activator of genes involved in embryo maturation and cellular differentiation [reviewed in [18]], was found to be differentially expressed in DGAT lines. Probes for WRI1, FUS3, ABI3, and ABI5 were present on the array but not differentially expressed in the DGAT lines, while probes for *GLABRA2 *and *LEC2 *were not present on the microarray.

Some of the genes previously reported to be differentially expressed in *B. napus *NILs differing in seed oil content [21] were also differentially regulated in DGAT transgenic lines. The list includes two genes identified as being abundant in the low seed oil containing *B. napus *NIL (Kelch repeat containing protein – At5g48180, and elongation factor – At5g19510) that were also down-regulated in DGAT transgenic lines and three other genes (putative 3-ketoacyl-CoA reductase – AY196196, pyruvate kinase – At5g52920, and a putative hydroxymethylbilane synthase – At5g08280) identified from the high seed oil NIL were up-regulated in our transgenic lines.

### k-PCR analysis

We validated the expression patterns of a number of genes through a different method, k-PCR, to rule out possible artifacts of microarray analysis and to provide additional confidence that these transcripts are in fact differentially expressed in developing DGAT transgenic seeds. Primers for k-PCR were designed based on available *Brassica *EST sequences and their expression levels were determined as a ratio of number of mRNA molecules between the transgenic and untransformed seeds per microgram of starting RNA used for cDNA synthesis.

**Figure 3:**
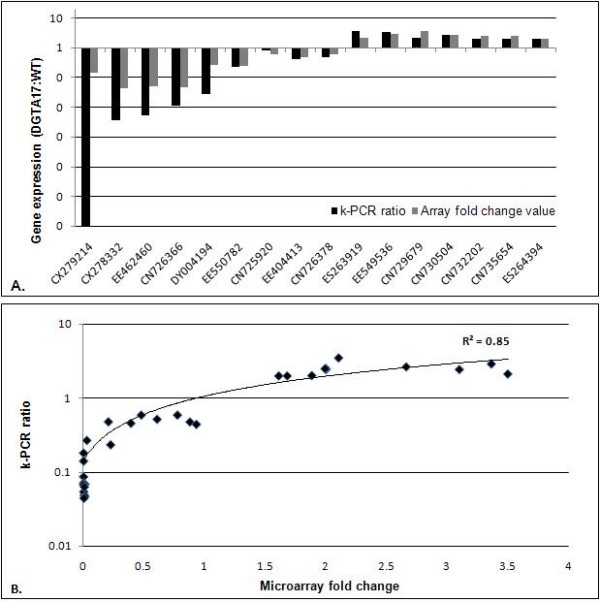
**Comparison of expression of representative genes selected from microarray data with k-PCR**. **(A) **Comparison of fold-change values from microarray data with expression ratios calculated from k-PCR. All values represent the average of 3 biological replicates, each analyzed twice (technical replicates). k-PCR, kinetic reverse transcriptase PCR; WT, untransformed control (*cv*. Quantum). **(B) **Correlation coefficient between the fold-change values from microarray and the expression ratios calculated from k-PCR presented as log10 of the ratio of gene expression in DGAT#17. k-PCR, kinetic reverse transcriptase PCR.

Differential expression of sixteen genes found to be up- or down-regulated by microarray was validated by k-PCR (Figure 3A). Results of this analysis correlated well with the data obtained from the microarray experiments, with an overall correlation coefficient of 0.85 between the microarray fold-change values and the expression ratios from k-PCR (Figure 3B). The relatively high level of correlation between our microarray results and k-PCR data supports the validity of this microarray platform in revealing gene expression changes.

**Figure 4:**
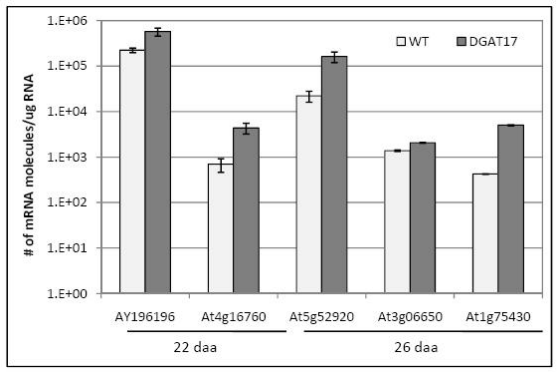
**Expression of genes previously correlated with seed oil content in transgenic line DGAT#17 and untransformed controls**. These genes were previously identified from *B. napus *near-isogenic lines differing in seed oil content. All values represent the average of 3 biological replicates, each analyzed twice (technical replicates) ± SE. 22 daa and 26 daa; gene expression examined at 22 and 26 days after anthesis, respectively; WT, untransformed control (*cv*. Quantum).

Using k-PCR, we also examined expression of five genes found to be differentially expressed between NILs of *B. napus *differing in seed oil content by 10% [21] and showed that these genes are also differentially expressed in DGAT#17 at one or more time points considered (Figure 4). The gene encoding a putative pyruvate kinase (At5g52920) was found to be the most highly differentially regulated in DGAT#17 followed by acyl-CoA oxidase (At4g16760) and putative 3-ketoacyl-CoA reductase 1 (AY196196). Genes encoding a homeodomain-like transcription factor (At1g75430) and ATP-citrate lyase (At3g06650/DY00115) were also found to be up regulated in DGAT#17 at 22 daa or 26 daa (Figure 4).

**Figure 5:**
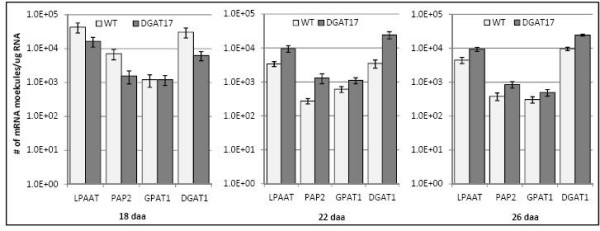
**Expression pattern of genes implicated in lipid-related processes in transgenic line DGAT#17 and untransformed control**. All values represent the average of 3 biological replicates, each analyzed twice (technical replicates) ± SE. 18 daa, 22 daa and 26 daa; Gene expression examined at 18, 22 and 26 days after anthesis, respectively. GPAT, glycerol-3-phosphate acyltransferase; LPAAT, lyso-phosphatidic acid acyltransferase; PAP, phosphatidate phosphatase; DGAT, diacylglycerol acyltransferase; WT, untransformed control (*cv*. Quantum).

Probes corresponding to a number of lipid-related genes including *BnDGAT1 *and genes from the Kennedy pathway are not present on the microarray platform used and thus their expression in DGAT#17 seeds was analyzed by k-PCR. As shown in Figure 5, genes encoding enzymes of the Kennedy pathway are differentially expressed in developing seeds of DGAT#17. Compared to untransformed controls, most of the genes encoding Kennedy pathway enzymes appeared to be slightly down-regulated in DGAT#17 at18 daa, but were subsequently up-regulated showing a progressively higher level of expression than in untransformed seeds over the next two developmental time points considered. Over the time course analyzed,*GPAAT *showed only a modest differential change in DGAT#17 relative to untransformed controls, whereas *DGAT1 *showed a strong differential expression change, as did *PAP2*.

### Some differentially expressed genes are responsive to plant hormones, especially ABA

**Figure 6:**
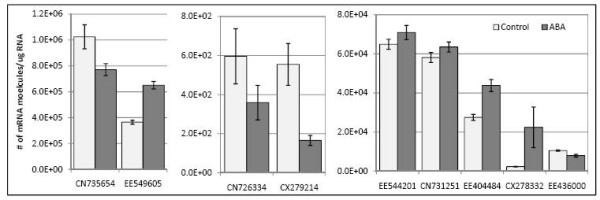
**Expression of novel transcripts in microspore derived embryos following treatment with ABA**. Early cotyledonary stage embryos were treated with 10 μM ABA (± racemic mix 1:1) in 0.1% ethanol or 0.1% ethanol without the hormone (Control) for 6 hours. Embryos were harvested, RNA extracted and analyzed for expression of a subset of novel genes identified from microarrays using k-PCR. All values represent the average of 2 biological replicates, each analyzed four times (technical replicates) ± SE.

As a first step in the functional characterization of the novel Brassica transcripts differentially expressed in DGAT transgenic lines, we tested whether their expression was responsive to ABA. Microscope derived embryos (MDEs) of *B. napus *were treated with ABA for 6 h and analyzed by k-PCR for expression of nine genes considered to be novel or having unknown function based on *Arabidopsis *annotation (Figure 6). Seven of the nine genes tested were responsive to ABA; three were up-regulated while four were down-regulated. Transcripts of CX278332, which was among the most highly down-regulated genes in DGAT transgenic seeds (Table 1), displayed close to 10-fold increase in response to ABA, while levels of CX279214 transcripts were reduced by close to 5-fold (Figure 6). These results indirectly suggest that the genes analyzed may have a role in seed maturation processes.

We also compared the list of genes found to be differentially regulated in DGAT transgenics with genes previously found to be regulated by plant hormones in the literature [38] and using the Meta Analyzer tool in Genevestigator (Genevestigator.com). Based on annotations derived from *Arabidopsis*, 2128 hormones related genes were found on the cDNA microarray, about 3.5% of which (69) were differentially expressed in DGAT transgenic lines. Of these 69 hormone responsive genes, 63 were found to be regulated by ABA and the remaining small numbers of genes were regulated by IAA, by GA or by cytokinin. Furthermore, the most substantial changes in expression occurred in response to ABA while the responses to other hormones were more or less subtle. Overall, 16 genes were identified as being responsive to one or more plant hormones at 18 daa and the number increased to 40 and 33 by 22 daa and 26 daa. Genevestigator database searches also revealed that several lipids-related genes differentially expressed in DGAT lines were responsive to ABA. Of note were genes encoding lipid binding/transfer proteins which were highly induced by ABA treatment. Two probes map to a common gene (At4g26740) in *Arabidopsis *which is similar to a rice ABA-responsive gene preferentially expressed in the embryo and is annotated as being involved in calcium ion binding . Together, these results suggest a potential link between DGAT overexpression and ABA-regulated gene expression. This is consistent with previous reports that ABA regulates lipid-related genes such as *OLEOSIN*, *FAD3 *and *FAE1 *[23,24,39].

### Hormone profiling of transgenic *DGAT *plants

Plant hormones are key regulators of many seed processes, including TAG accumulation. Accordingly, we profiled major classes of plant hormones (ABA, auxins, cytokinins, and GA) throughout the period of seed oil deposition (14 daa to 34 daa). Hormone metabolites were also quantified because many have been shown to be biologically active and analysis of several related metabolites provides information on the flux through pathways [24]. Hormones were analyzed by HPLC-MS/MS using multiple reactions monitoring and deuterated internal standards for each of the hormones and metabolites. Levels of GA were generally too low to allow meaningful comparisons to be made between untransformed and transgenic seeds (data not shown).

#### a) ABA

**Figure 7:**
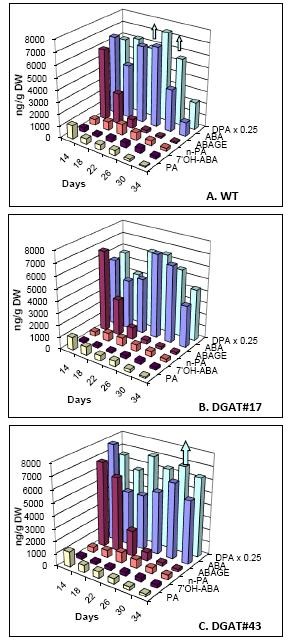
**Levels of ABA and metabolites in seeds of untransformed control and two independent transgenic lines**. **(A) **WT, untransformed control (*cv*. Quantum); **(B) **DGAT#17; **(C) **DGAT#43. DPA 0.25, dihydrophaseic acid (measured levels divided by four); ABAGE, ABA glucose ester; 7'-OH ABA, 7'-hydroxy ABA; *n*-PA, *neo*-phaseic acid; PA, phaseic acid. Upward arrows on bar graph for DPA, seed DPA levels at that time point were beyond detection limit of our method. Values represent averages of three biological replicates.

We have recently published the profile of ABA and the catabolites 7'-OH ABA, phaseic acid (PA), dihydrophaseic acid (DPA), *neo*-phaseic acid (*n*-PA) and ABA glucose ester (ABA-GE) in seeds of untransformed *cv*. Quantum [24], but include the information here to allow comparison with the transgenic lines. As the equilibria between open and closed forms can change during extraction and analysis, the PA and *n*-PA levels represent the total of hydroxylated product at either the 8'- or 9'-position, respectively. In untransformed seeds, two peaks of ABA are observed (Figure 7), similar to the case in *Arabidopsis *[40]. DPA was the most abundant ABA metabolite detected, with its levels peaking at 26 daa. Pools of ABA-GE at 14 daa are as high as those of ABA, but thereafter rapidly declined. The remaining ABA metabolites were present at substantially lower levels. Transgenic seeds displayed reduced levels of ABA at 22 daa, but generally contained higher levels of ABA and DPA at later time points (30 and 34 daa). No substantial changes were detected in pools of the remaining ABA metabolites.

#### b) Auxins

**Figure 8:**
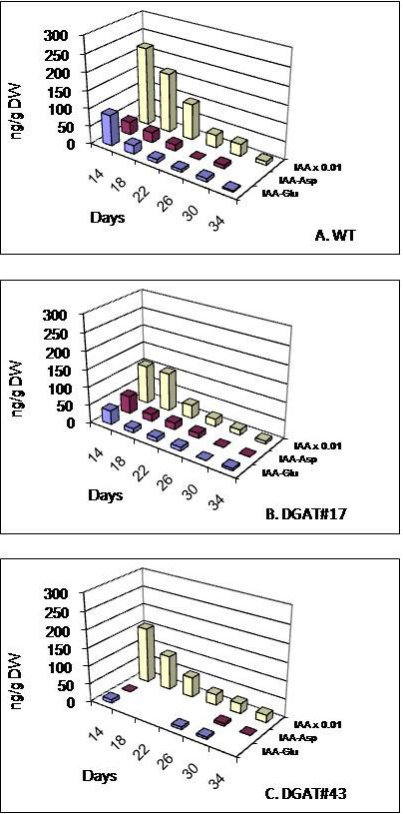
**Levels of auxins in seeds of untransformed control and two independent transgenic lines**. **(A) **WT, untransformed control (*cv*. Quantum), **(B) **DGAT#17, **(C) **DGAT#43. IAA, Indole acetic acid (measured levels divided by 100); IAA-Asp, IAA aspartate; IAA-Glu, IAA glutamate. Values represent averages of three biological replicates.

In seeds of untransformed plants, indole acetic acid (IAA) was the predominant auxin detected (Figure 8). Its level was highest at 14 daa and gradually declined thereafter. Two amino acid conjugates of IAA, IAA-aspartate and IAA-glutamate, were also detected at very low levels sporadically throughout development. In transgenic seeds, levels of IAA was initially higher (14 daa) in DGAT#17, but not in DGAT#43. At subsequent times, IAA levels between untransformed and transgenic seeds were comparable. Levels of the two IAA conjugates were consistently higher in seeds of both transgenic lines, especially early in development.

#### c) Cytokinins

**Figure 9:**
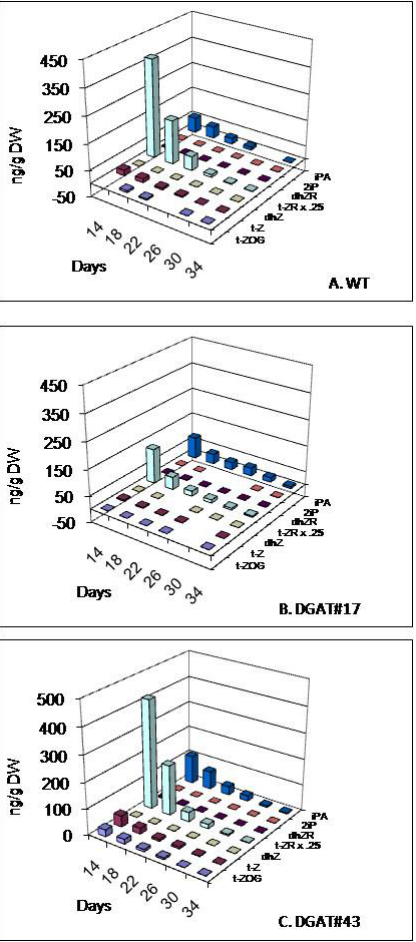
**Levels of Cytokinins and metabolites in seeds of untransformed control and two independent transgenic lines**. **(A) **WT, untransformed control (*cv*. Quantum), **(B) **DGAT#17, **(C) **DGAT#43. t-ZOG, *trans*-zeatin-O- glucoside; t-Z, *trans*-zeatin; dhZ, dihydrozeatin; t-ZR,*trans*-zeatin riboside (measured levels divided by four); dhZR, dihydrozeatin riboside; 2iP, 2-isopentenyl adenine; iPA, isopentenyl adenosine. Values represent averages of three biological replicates.

*trans*-zeatin ribose (t-ZR) was the predominant cytokinin detected in *B. napus *seeds, followed by isopentenyl adenosine (IPA) and *trans*-zeatin (t-Z) (Figure 9). Levels of these cytokinins were highest at the earliest time sampled (14 daa) and gradually declined. Cytokinin levels were essentially the same in untransformed and transgenic seeds, with the possible exception of IPA, which was slightly higher in DGAT#43 at 14 daa.

## Discussion

DNA microarrays have been successfully used to examine changes in gene expression associated with transgenesis. Analysis of plants expressing selectable and marker transgenes has revealed that stable insertion of T-DNA within the plant genome *per se *has little effect on the transcriptome [41]. In fact, it has been proposed that transgenesis yields fewer changes in gene expression than either mutagenesis [42] or conventional breeding [43]. However, expression of transgenes encoding plant enzymatic or regulatory functions can interfere with the normal expression patterns of corresponding endogenous genes [44] as well as genes involved in the same or related processes [42].

Our results of transgenic *B. napus *plants expressing *AtDGAT1 *are consistent with the above reports. Overall, only modest changes in the transcriptome and in the levels of several of the hormones analyzed were detected between transgenic seeds and untransformed controls. Many of the genes found to be differentially regulated appear to be involved in lipid- or membrane-related processes. This conclusion is based on GO annotations and follow-up k-PCR analysis. Of note, genes encoding enzymes of the Kennedy pathway were shown to be up-regulated in the transgenic seeds. Furthermore, the remarkably high level of differential expression of the gene encoding a putative pyruvate kinase and a homeodomain transcription factor in transgenic seeds indicates that DGAT over expression may also affect upstream metabolic pathways contributing to the biosynthesis of fatty acids, to be incorporated into TAGs. It has been suggested previously that TAG biosynthesis could be regulated by upstream metabolic processes such as the supply of carbon in the form of fatty acids [16]. Together, our data suggest that increasing DGAT activity affects a series of genes involved in lipid metabolism, possibly through feedback or feed-forward effects. Similarly, a significant number of differentially expressed genes between two *B. napus *NILs differing in oil content were found to be related to metabolic processes [21]. It was suggested that these genes may be important in seed development for the storage of reserve materials. Several of the genes identified by Li et al. [21] are also differentially expressed in the DGAT transgenic analyzed in the current study suggesting that common mechanisms may underlie the increases in seed oil content in both plant material.

The fact that more than 50% of these represent novel transcripts with no counterpart in other organisms or are related to *Arabidopsis *genes annotated as "unknown function" compromised our ability to extract additional information from the data but could eventually provide new insights into TAG biosynthesis in *Brassica*. Validation of microarray results through k-PCR ruled out the possibility that the differential expression patterns of the novel transcripts were artifacts. Since microarray hybridization and k-PCR are technically very different in many aspects including methods of normalization, sensitivity and specificity, a wide range of correlations (-0.48 to 0.94) between data generated by these methods have been reported [45]. Of note, our microarray protocol included an RNA amplification step, which was excluded in the k-PCR analysis. The relatively high level of correlation (0.85) between our microarray results and k-PCR data supports the validity of our microarray analysis in revealing gene expression changes.

Many of the enzymes involved in lipid biosynthesis, including those of the Kennedy pathway, are encoded by multigene families. The relative importance of different enzyme isoforms within these families towards seed oil biosynthesis in *B. napus *is currently not well understood. Accordingly, it is possible that our microarray and/or k-PCR transcript profiling may not have considered the most relevant gene family members. Furthermore, our experimental design does not rule out the possibility that increased seed oil achieved through transgenic overexpression of DGAT1 is mediated, at least in part, by effects at the translational and/or post-translational levels. Additional research, including targeted analysis of different isoforms of lipid biosynthetic enzymes, and global, proteome-wide characterization of proteins affected by DGAT1 overexpression will be required to answer the above questions.

Overexpression of DGAT1 also resulted in changes in the profiles of major hormone classes, including auxins, cytokinins and ABA. As observed with transcript profiling, overall changes in hormone levels appeared to be relatively modest and no clear correlations between levels of different hormones and oil content were apparent. However, *in silico *analyses indicated that several genes identified in the DGAT transgenic seeds during oil synthesis phase are inducible by plant hormones, especially ABA, a hormone known to regulate the maturation phase of seed development. Analysis of novel transcripts by k-PCR also suggested that they are also rapidly inducible by ABA. The extent to which altered ABA levels contribute to the differential accumulation of these and other genes in the transgenic plants, and the mechanism by which increased DGAT activity results in altered seed ABA levels are currently unknown. It is possible that the functional characterization of the novel transcripts identified in this study could shed light on these questions. Further analysis of these novel transcripts is warranted, given their dramatic changes in expression in seeds of transgenic DGAT plants throughout time course of study and their rapid response to exogenous ABA. The knowledge obtained could eventually be applied to engineer Brassica crops with higher seed oil contents.

## Conclusion

Global transcript profiling of seeds during the period of active oil biosynthesis identified a number of changes associated with transgenic overexpression of DGAT. These included increased steady-state levels of genes encoding endogenous BnDGAT1, enzymes of the Kennedy pathway and other lipid-related processes, suggesting that altering DGAT1 levels affects several steps upstream in the fatty acid and TAG biosynthetic pathways. This confirms previous speculation [12] that increased DGAT levels may result in secondary regulatory effects. Determining the specificity of gene expression changes was not possible given the large fraction of differentially regulated transcripts having no counterparts in other organisms, thus compromising our ability to draw concrete inferences from the data. Nonetheless, this study has led to the identification of novel Brassica transcripts of potential interest. Ascertaining whether and how these are involved in seed development, and particularly lipid biosynthesis, will require further study. The identification of a common set of genes differentially expressed in DGAT transgenics and Brassica NILs differing in oil content is also noteworthy as these may serve as markers for oil content. It will be interesting to ascertain whether these genes are also differentially expressed in transgenic material engineered to accumulate higher oil levels through other strategies, such as overexpression of *sn*-2 acyltransferase [46], glycerol-3-phosphate dehydrogenase [47] or DOF transcription factors [19], or suppression of mtPDCK [16].

## Methods

### Plant growth

*Brassica napus *plants (*cv*. Quantum and transgenic DGAT lines #17 and 43) were germinated on moist filter paper in the dark. Seedlings were transferred to soil (Readi-Earth) in an environmentally controlled cabinet with a 16 h day (22°C; 175 μE)/8 h night (17°C) photoperiod and watered daily with a 1:10 dilution of 20:20:20 (nitrogen:phosphorous:potassium) fertilizer. Flowers on the primary and secondary inflorescences were self-pollinated by hand at anthesis and tagged. Developing seeds were dissected from siliques on dry ice at 14, 18, 22, 26, 30, and 34 days after anthesis (daa), frozen immediately in liquid nitrogen, and stored at -80°C until analyzed.

### Analysis of DGAT activity and seed traits

Assays of DGAT activity were performed on microsomal fractions from developing seeds at 26 daa as previously described [12]. Briefly, acyl-CoA-dependent DGAT activity assays were conducted at pH 7.4 with shaking at 100 rpm in a water bath at 30C for 30–60 min. Assay mixtures (500 ml final volume) contained microsomal protein (100–200 mg), 90 mM HEPES-NaOH, 0.5 mM ATP, 0.5 mM CoASH, 1 mM MgCl2, 200 μM *sn*-1,2 diolein in 0.02% (v/v) Tween 20, and 18 μM [1-^14^C]-18:1-CoA. Reactions were terminated and ^14^C triacylglycerols were isolated, purified by TLC and quantified.

Seed oil contents and quality traits were measured according to Taylor et al. [14]. Oil content of mature seed from *B. napus *(cv Quantum) was determined by low-resolution nuclear magnetic resonance spectroscopy (LR-NMR) using a Bruker Minispec mq20 instrument, calibrated with mature *B. napus *seed of known oil content obtained from the Grain Research Laboratory of the Canadian Grain Commission (Winnipeg, MB, Canada). All seed quality data collected from each line were analyzed with the Minitab Statistical Software Suite Release 12 (Minitab, State College, PA 16801–3008) using the Anova-Fisher's LSD method (p ≤ 0.05) and Tukey's method of pairwise comparisons of all transgenic lines to the non-transformed Quantum control.

### RNA extraction, aRNA amplification, and fluorescent labeling of aRNA

Microscope derived embryos (MDEs) were grown and total RNA isolated as previously described [24]. Total RNA was isolated from seed using Agilent Plant RNA isolation minikit (Agilent Technologies Canada Inc., Mississauga, ON). Samples (25 mg FW) were ground into a fine powder in liquid nitrogen and total RNA was isolated according to manufacturer's instructions. Each total RNA sample (5 μg) was converted to amplified RNA (aRNA) using Amino Allyl MessageAmp™ II aRNA Amplification Kit (Ambion Inc./Applied Biosystems Canada Streetsville, Ontario) and 20 μg each aRNA samples were labeled using the Cyscribe Post-Labeling kit (GE healthcare Life Sciences Inc., Baie d'Urfé, Québec) following the manufacturer's instructions. The CySribe GFX Purification kit (GE healthcare Life Sciences Inc., Baie d'Urfé, Québec) was used to purify the fluorescent dye-labeled cDNA probe by removing free nucleotides and unincorporated CyDye molecules.

### Hybridization and washing

*Brassica *10 K cDNA microarrays (average probe length of approximately 489 bases) were synthesized at BRI/NRC as described in Xiang et al. [31]. The slides were prehybridized at 37°C for a minimum of 1 h. The prehybridization buffer was rinsed off by placing the slide in a 50-ml tube for 2 min, first in H_2_O, then in 70% ethanol, and then in 100% ethanol. The slide was then air dried. Probe/hybridization buffer (62.8% formamide, 0.8% SDS, 4× Denhardt's, 5× SSPE) was denatured at 95°C for 3 min. Then probe solution was placed over the pre-hybridized slide. A coverslip (Sigma) was applied to the slide and hybridization was performed overnight in a water bath at 42°C. During both the pre-hybridization and hybridization steps, raised cover slip configurations were used. Four to seven biological replicates were performed for each experiment, half of which were dye-swapped.

After hybridization, each slide was washed with 200 ml 2× SSC/0.1% SDS once at 42°C for 4 min, once with 0.2× SSC/0.1%SDS for 4 min at room temperature, and then twice with 0.2× SSC at room temperature for 2.5 min each. Slides were placed in a 50-ml tube and spun in a swinging bucket rotor centrifuge for 5 min at 1000 rpm to dry.

### Image analysis, normalization and data analysis

Arrays were scanned at 10 μm resolution in a ScanArray 4000 scanner (PerkinElmer, Woodbridge, ON). In several experiments, two scans were performed for each array: one using high laser and gain settings to avoid the non-linear effects at lower intensities affecting low-intensity spots and background estimates; and one using low laser and gain settings to obtain correct estimates for the strong spots which will contain saturated pixels in the "high" scan. Spot location and intensity quantitation were performed using QuantArray version 3.0 (PerkinElmer). Histogram spot quantitation, known to give the lowest between-slide variability [48], was employed and median intensity values were used for subsequent analysis.

Localized background subtraction and merging of the intensity values from "high" and "low" scans was performed using the BASE software platform [49]. The resulting signals from each channel were normalized using shrunk robust splines (Gordon Smyth, unpublished) available through the Bioconductor (Limma) and SMA tools in the R statistics package [50]. Log_2 _transformed paired channel intensity values (background subtracted and normalized) from replicate hybridizations were subjected to Significance Analysis of Microarrays (SAM) software [51] to identify statistically significant up- or down-regulated genes. Delta values were adjusted to achieve a false discovery rate as close to 5%. Significant genes were mapped to the *A. thaliana *gene database () for their annotation information. Genes exhibiting significant changes in expression were also grouped into different functional categories based on gene ontology (GO). GeneSpring Software (Agilent Technologies) was used to explore expression patterns and/or to compare the SAM-generated lists of significant genes with the literature. A list of genes identified from significant analysis of microarrays, and all raw data are presented in Additional Files 1 and 2a–2d.

### Kinetic polymerase chain reaction

Kinetic reverse transcriptase Polymerase Chain Reaction (k-PCR) was performed on an MX3000 spectrofluorometric thermal cycler (Stratagene, LaJolla, CA) using a two temperature cycling regime initiated with a 15 min activation at 95°C, followed by 40 cycles of 2 min of annealing and extension at 66C or 62°C, depending on primer pairs, and 10 sec denaturation at 95°C. Each assay contained 0.5 pmol oligonucleotides, 5 ng cDNA, and 1 × SYBR Green^® ^(Quantitech; Qiagen, Mississauga, ON), prepared as described in Rutledge and Stewart [52]. The fluorescence data collected at the end of each PCR cycle was analyzed by the absolute quantification via C_t _method [52].

### Quantification of plant hormones by LC-MS/MS

The procedures for extraction and quantification of plant hormones in seeds of untransformed and transgenic DGAT plants were similar to those described in Chiwocha et al. [53]. Synthesis of the deuterium-labeled ABA internal standards was described by Abrams et al. [54] and Zaharia et al. [55].

## Authors' contributions

NS and MA performed gene expression studies, including microarrays and k-PCR. NS also helped conceive these studies, analyzed and interpreted the data, and prepared corresponding figures. AK and YZ generated the transgenic plants, analyzed them for transgene presence and expression, and seed weight, including growth cabinet and field trials. EMG performed and interpreted lipid analyses and DGAT assays. SRA and LIZ performed and interpreted the hormone profiling, and prepared corresponding figures. DCT conceived and coordinated the production and analysis of the transgenic plants. NS and PF drafted the manuscript with the assistance of SRA, LIZ and DCT. SRA, DCT and PRF conceived the study as a whole. PRF also co-ordinated and supervised the project. All authors read and approved the final manuscript.

## Supplementary Material

Additional File 1 Significant Genes. List of 197 significant genes identified from both DGAT lines, DGAT#17 and DGAT#43. Click here for file

Additional File 2 **a–2d**. Raw Intensities. Raw intensity values for Cy3 and Cy5 channels from all hybridizations between untransformed control (*cv*. Quantum) and DGAT transgenic seeds. Click here for file
